# Large language models for risk-of-bias assessment in randomised clinical trials—a comparative validation study

**DOI:** 10.1016/j.ebiom.2026.106238

**Published:** 2026-03-28

**Authors:** Lauri Nyrhi, Ville Ponkilainen, Juho Laaksonen, Lauri Kuikka, Lauri Paljakka, Teemu Karjalainen, Ville M. Mattila, Ilari Kuitunen

**Affiliations:** a Department of Orthopaedics and Traumatology, Tampere University Hospital, Finland; b Faculty of Medicine and Health Technology, Tampere University, Finland; c Department of Surgery, Central Finland Hospital Nova, Jyväskylä, Finland; d Department of Hand- and Microsurgery, Tampere University Hospital, Finland; e Institute of Clinical Medicine, University of Eastern Finland, Kuopio, Finland; f Department of Pediatrics, Kuopio University Hospital, Kuopio, Finland

**Keywords:** Risk of bias, Large language model, Methodology, Artificial intelligence

## Abstract

**Background:**

Large language models (LLMs) are emerging tools for evidence synthesis. Risk of bias (RoB) assessment of trials remains an essential but time-consuming step inconsistent even amongst experts. Early LLM studies showed mixed reliability. Advances in reasoning-enabled models warrant evaluation of their accuracy and consistency for RoB screening across randomised trials to reduce reviewer workload.

**Methods:**

We conducted a preregistered comparative validation study (March 11–May 19, 2025) of four LLMs—ChatGPT o3, DeepSeek v3, Google Gemini Flash 2.0, and Grok 3—prompted with full-text randomised clinical trial articles and protocols. Two corpora were analysed: 100 RCTs from recent Cochrane reviews (RoB 1) and 100 RCTs from meta-analyses in high-impact journals (RoB 2). The reference standard was published human RoB judgements. The primary outcome was interobserver reliability (Cohen κ, 95% CI); secondary outcomes were intraobserver agreement and diagnostic accuracy (sensitivity, specificity, predictive values, F_1_-score).

**Findings:**

For RoB 1, interobserver agreement ranged from κ 0.0.27 (95% CI 0.07–0.46) with Gemini Flash 2.0 to κ 0.39 (0.20–0.59) with DeepSeek v3. For RoB 2, agreement was lower, from κ 0.06 (−0.07 to 0.18) with ChatGPT o3 to κ 0.13 (−0.04 to 0.31) with Gemini. Diagnostic performance was limited with sensitivity ranging 0.05–0.55, specificity 0.78–0.99, PPV 0.31–0.50, and NPV 0.48–0.61 across models, with models consistently over-flagging concerns.

**Interpretation:**

None of the evaluated LLMs were sufficiently reliable for fully autonomous RoB assessment. DeepSeek v3 and ChatGPT o3 approximated human performance best on RoB 1, but RoB 2 rule-in and rule-out performance remained modest. Current use should be supervised, with possible application of LLMs for triage or as a second assessor. Major improvements in protocol retrieval, task-specific tuning, and calibrated thresholds, prospectively validated, are needed for safe stand-alone deployment.

**Funding:**

This study received no financial support.

**Research in context**

**Evidence before this study**

We conducted a focused search of PubMed, arXiv, and medRxiv up to May 19, 2025, using terms such as “large language model,” “ChatGPT,” “risk of bias,” “Cochrane,” and “diagnostic accuracy,” and reviewed reference lists of relevant reports. Prior studies evaluating LLMs for Cochrane risk-of-bias judgements were few with typically small sample sizes. Findings were mixed: some reported promising performance, others poor agreement with human reviewers, and most did not assess RoB 2 comprehensively, examine domain-level reliability, or include intraobserver consistency. We found no preregistered, comparative evaluation of multiple contemporary LLMs using full texts across specialities.

**Added value of this study**

In a preregistered diagnostic accuracy and reliability study of four advanced LLMs (ChatGPT o3, DeepSeek v3, Grok 3, Gemini Flash 2.0) applied to 200 randomised trials (100 RoB 1; 100 RoB 2; 2600 domain judgements), interobserver agreement with human assessors was fair for RoB 1 and poor for RoB 2, however matching human values. For RoB 2, models over-flagged trial bias, yet had limited rule-in and rule-out performance, supporting supervised triage rather than autonomous use.

**Implications of all the available evidence**

The findings or our study verify that contemporary LLMs are not yet suitable for autonomous risk-of-bias assessment. They often flag concerns when risk is low and struggle to reliably confirm either clearly high- or clearly low-risk trials, especially with RoB 2. At present, the most appropriate use is with human oversight prioritising studies for manual review or acting as a second assessor. Such use could potentially reduce workload and accelerate evidence synthesis. Safe deployment will require task-specific tuning, automated retrieval of trial protocols/registrations, clear preset operating thresholds, and prospective validation with ongoing monitoring.

## Introduction

Artificial intelligence (AI) encompasses computer algorithms capable of tasks traditionally requiring human intelligence, including data interpretation, decision-making, and problem-solving. Among these, large language models (LLMs) have rapidly emerged as transformative tools, analysing extensive textual data to generate human-like text based on statistical patterns learnt from diverse datasets. In healthcare research, LLMs hold considerable promise for automating resource-intensive processes, such as systematic reviews and meta-analyses.

Previous research evaluating LLM performance, particularly for complex cognitive tasks like Risk of Bias (RoB) assessments in systematic reviews, has shown mixed results.^1^, ^2^, ^3^, ^4^ Non-reasoning LLMs such as ChatGPT demonstrated promising but insufficient reliability, while Lai et al. reported excellent yet unreplicated accuracy.^5^ However, human evaluations are also subject to limitations. Inherent biases and suboptimal interobserver agreement have been documented even within top-tier medical journals. These challenges underscore the potential value of AI tools in complementing or enhancing the consistency and efficiency of bias assessments in evidence synthesis.^6^, ^7^, ^8^, ^9^

Recent advancements have produced reasoning-enabled LLMs that incorporate structured cognitive approaches, such as chain-of-thought (CoT) prompting. These models perform better on complex evaluation tasks, including benchmarks like Humanity's Last Exam (HLE), suggesting enhanced reasoning capacity and methodologic potential.^10^ Given these developments, reassessing the performance of LLMs in RoB assessments is warranted. Currently, RoB evaluations using established tools like Cochrane's RoB 1 and RoB 2 necessitate considerable human expertise and effort, representing a substantial resource burden.^6^^,^^7^ Although interest in AI integration is increasing, thorough comparative studies evaluating advanced reasoning-based LLMs against expert human judgement are still missing. If reliable, such models could streamline systematic review workflows by reducing time and resource demands while maintaining methodological rigour. This may help narrow the gap between evidence generation and clinical implementation.

To address this gap, we evaluated the performance of four commercially and publicly available advanced LLMs—OpenAI ChatGPT o3, DeepSeek v3, Grok 3, and Google Gemini Flash 2.0—in conducting RoB assessments on published randomised controlled trials. The study assessed both interobserver reliability between AI- and human-generated evaluations and intraobserver reliability (consistency within AI-generated assessments). Ratings used the Cochrane RoB tools, specifically the RoB 2 (2019) and RoB 1.0, which evaluate methodological risk of bias in randomised trials and yield domain-level and overall judgements, applied by human experts and LLMs.^11^^,^^12^ To date, most published meta-analyses have used the original RoB 1 tool, whereas reviews applying RoB 2 have only recently begun to appear. As the first head-to-head evaluation of multiple contemporary LLMs, we included both RoB tools but focused interpretation on RoB 2. These findings are intended to report the feasibility and limitations of incorporating advanced LLMs into systematic review workflows.

## Methods

### Study design

This comparative reliability study assessed the accuracy of LLMs in performing RoB assessments compared with human experts. Data were collected between 11 March 2025 and 19 May 2025. The protocol was preregistered on the Open Science Framework (DOI 10.17605/OSF.IO/K45C7) and is reproduced in eAppendix 1.

### Data sources and selection criteria

For RoB 1, we included the most recent Cochrane reviews available in orthopaedics, pediatrics, oncology, and cardiology as of 11 March 2025. Reviews were eligible if they contained at least 10 randomised controlled trials (RCTs) with publicly available full texts and RoB assessments. From each eligible review, the alphabetically first 10 RCTs by author were selected, totalling 100 RCTs. For RoB 2, we included meta-analyses published in The Lancet, JAMA, Annals of Internal Medicine and the BMJ. We selected chronologically the first meta-analyses containing at least five RCTs and included alphabetically the first 10 studies per meta-analysis until we totalled 100 RTCs. Human expert-generated RoB assessments were obtained from the original Cochrane reviews (RoB 1) and published meta-analyses (RoB 2), all of which adhere to high methodological standards for RoB assessment and were used as the gold standard reference for accuracy parameters. We did not audit the internal assessment procedures of each review. The full RoB extraction dataset was independently checked by a second author against the source reviews and meta-analyses and no discrepancies were found.

### Large language models evaluated

The evaluated LLMs were ChatGPT o3 (OpenAI, May 2025 build),^13^ DeepSeek v3 (DeepSeek, April 2025),^14^ Grok 3 (xAI, April 2025),^15^ and Gemini Flash 2.0 (Google, April 2025).^16^ Model IDs, token limits, context window, interface used and access dates are listed in eTable 1. All models were used in their base mode though web chat with no additional features enabled.

### Prompt development and standardisation

We adapted a standardised prompt from Lai et al.^5^ The full prompt text is provided in eAppendix 2. PDF full texts of each RCT with PDF-versions of their protocols when available were uploaded directly into each LLM, except for Deepseek v3, as detailed below in the Deviations from Protocol section.

### Assessment procedures

Cochrane Risk of Bias 1.0 tool (RoB 1) classifies studies into one of three-categories: high risk, unclear risk, or low risk of bias. Cochrane RoB 2 tool classifies studies as high risk, some concerns, or low risk of bias. Reviewed domains for each RoB tool are specified in eTable 2. To assess interobserver reliability, each LLM independently completed a single RoB assessment for each included RCT and each assessment was compared with the corresponding human expert assessment. For intraobserver reliability, each model repeated the assessments independently by a second author using a new session and account. RoB assessments were categorised for all seven RoB 1 domains and five distinct RoB 2 domains in addition to the overall domain, totalling 2600 assessments.

### Statistics

Agreement metrics and diagnostic accuracy estimates are summarised in Table 1. The primary outcome was interobserver reliability, measured with unweighted Cohen κ.^17^ As a secondary agreement measure for the primary outcome, we also calculated the Gwet second-order agreement coefficient (AC2).^18^ Intraobserver reliability was assessed with Cohen κ. All agreement estimates are reported with 95% CIs. To account for clustering within source reviews or meta-analyses, we performed a cluster bootstrap sensitivity analysis and calculated cluster-adjusted 95% confidence intervals for κ and AC2. The F_1_-score was calculated to capture the balance between precision and recall for trials judged at high risk of bias. Diagnostic proportions (sensitivity, specificity, PPV, NPV, and F_1_) are presented as point estimates with 95% confidence intervals.^19^^,^^20^ For RoB 1, PPV was defined as any domain “High bias” per article and NPV for all domains “Low bias”. For RoB 2, PPV was calculated with similar definitions using solely the overall domain D6. Marginal homogeneity was assessed using the Stuart–Maxwell test with Benjamini–Hochberg false discovery rate adjustment.^21^^,^^22^ The sample-size calculation (n = 100 trials per RoB version) indicated 80% power to detect a κ difference of 0.15 (α = 0.05). A sensitivity analysis tested whether missing protocol information affected performance in Domain 5 (Selective outcome reporting); for trials without an available protocol, the gold-standard Domain 5 rating was imputed and the overall Domain 6 judgement recalculated. Data on sex was not collected or analysed as part of the study design. Analyses were conducted in R, version 4.4.2, using the tidyverse, irrCAC, irr, epitools, DescTools and patchwork packages. All code and data, including the full risk-of-bias extraction dataset, are available in the Open Science Framework (OSF) repository linked to the preregistered protocol (see Data Sharing Statement).

**Table 1:** Statistical parameters used to evaluate large language models.

Outcome/Statistic	Purpose	Key details
Primary outcome: Inter-observer agreement	Concordance between human and LLM ratings across all seven RoB 1 domains, and the overall RoB 2 domain (D6)	Unweighted Cohen κ (primary) and AC2 (secondary), with 95% Cls
Domain-specific agreement (D1–D5, RoB 2)	Granular agreement at domain level	Cohen κ with 95% CIs
Intra-observer agreement	LLM self-consistency on repeat assessments	Cohen κ with 95% CIs
Sensitivity, specificity, PPV	Ability to flag high-risk trials	Point estimate with 95% CIs
NPV	Ability to confirm low-risk trials	Point estimate with 95% CIs
Combined sensitivity	Detection of trials at high or some-concern risk	Point estimate with 95% CIs
F_1_-score	Harmonic mean of PPV and sensitivity	Point estimate
Directional bias counts	Whether LLM ratings skew higher/lower/equal to humans	Tally of counts
CI methods	Precision estimation	Large-sample normal (κ, AC2); Wilson (diagnostic)
Statistical tests & correction	Marginal homogeneity & multiple testing	3 × 3 Stuart–Maxwell; Benjamini–Hochberg FDR

**Abbreviations:** AC2, Gwet second-order agreement coefficient; CI, confidence interval; κ, Cohen kappa; LLM, large-language model; NPV, negative predictive value; PPV, positive predictive value; RoB, risk of bias; FDR, false-discovery rate.

### Ethics

Ethical approval or informed consent was not required, as the study did not involve human participants or any new primary data. The study adhered to ethical standards outlined in the Declaration of Helsinki.

### Reporting standards

This study followed Guidelines for Reporting Reliability and Agreement Studies (GRRAS) and CONSORT-AI guidelines, with full details provided in eAppendix 3 and eAppendix 4, respectively.

### Role of funders

This study received no financial support.

## Results

### Study selection and characteristics

For RoB 1 analysis, we included a total of 100 RCTs and 700 individual RoB assessments. Three reviews were from the field of orthopaedics and trauma,^23^, ^24^, ^25^ three were paediatric reviews,^26^, ^27^, ^28^ two were from oncology^29^^,^^30^ and two were from cardiology.^31^^,^^32^ All reviews were published between 2024 and 2025. For RoB 2 analysis, two reviews were from the field of internal medicine,^33^^,^^34^ two from vascular surgery,^35^^,^^36^ one in cardiology^37^ and one from pulmonary medicine.^38^ All reviews were published between 2023 and 2024. Across all models, low-risk judgements predominated, although the absolute distribution varied (eTable 3).

### Primary outcome: interobserver reliability

**Fig. 1:**
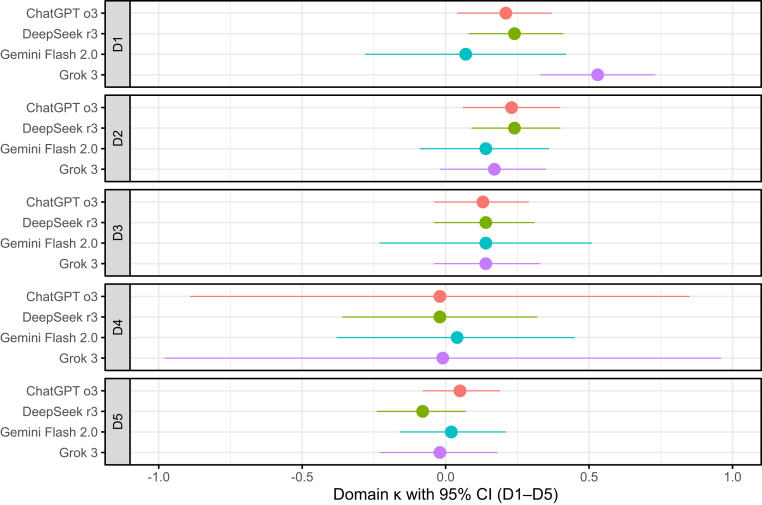
Domain-level inter-observer reliability in cohens Kappa (κ) of each large language model for risk of bias 2.

**Fig. 2:**
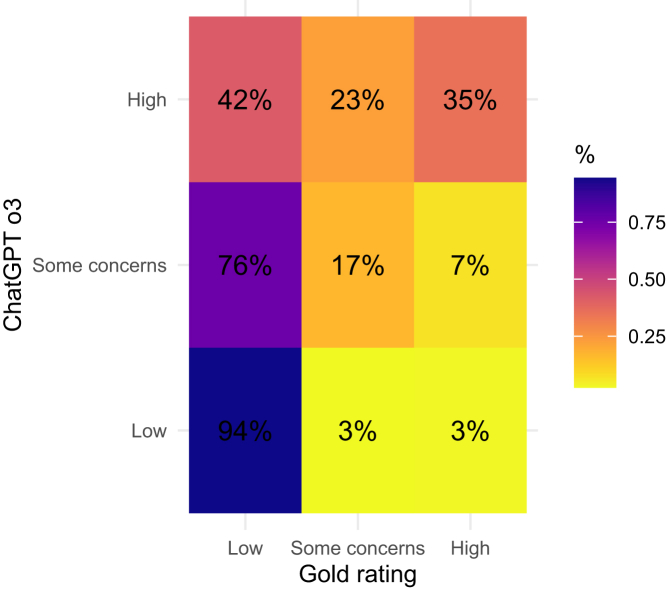
Pooled 3 × 3 Heat Map of ChatGPT o3 vs Gold-Standard Ratings for Risk of Bias 2.

Results for interobserver reliability comparing large language models with the human reference standard are summarised in Table 2. For RoB 1, κ ranged from 0.27 (95% CI, 0.07–0.46) for Gemini Flash 2.0 to 0.39 (95% CI, 0.20–0.59) for DeepSeek v3. AC2 values parallelled κ (range, 0.51–0.55; 95% CIs in Table 2). For RoB 2, interobserver reliability was lower, with κ ranging from 0.06 (95% CI, −0.07 to 0.18) for ChatGPT o3 to 0.13 (95% CI, −0.04 to 0.31) for Gemini Flash 2.0. Domain-specific RoB 2 κ values with 95% CIs are shown in Fig. 1. Discordance patterns for ChatGPT o3 are shown in a confusion matrix heat map (Fig. 2). Cluster-adjusted confidence intervals for κ and AC2 were similar to the primary estimates and did not change interpretation (eTables 4A and 4B).

**Table 2:** Inter-observer Agreement and Diagnostic Accuracy of the Four Large Language Models vs the Gold Standard.

Model	RoB 1 κ (95% CI)	RoB 2 κ (95% CI)
ChatGPT o3	0.36 (0.18–0.54)	0.06 (−0.07 to 0.18)
Google Gemini Flash 2.0	0.27 (0.07–0.46)	0.13 (−0.04 to 0.31)
DeepSeek v3	0.39 (0.20–0.59)	0.12 (−0.01 to 0.25)
Grok 3	0.28 (0.10–0.46)	0.09 (−0.05 to 0.22)

**Abbreviations:** CI, confidence interval; κ, Cohen kappa; RoB, risk of bias.

### Secondary outcomes

Intraobserver reliability and diagnostic accuracy metrics are summarised separately for RoB 1 (Table 3) and RoB 2 (Table 4). For RoB 1, intraobserver reliability ranged from κ 0.47 (95% CI 0.33–0.60; Gemini Flash 2.0) to κ 0.71 (95% CI 0.60–0.83; Grok 3). F_1_-scores were 0.82–0.92, indicating a balanced ability to identify high-risk studies and avoid false positives. Exact 95% confidence intervals for all RoB 1 diagnostic metrics are provided in eTable 5A, and domain-specific intra- and inter-observer κ values in eTable 6.

**Table 3:** Performance of large language models on risk of bias 1 assessment metrics.

Model	Intra-κ (95% CI)	Sensitivity	Sensitivity (High/Unclear)	Specificity	PPV	NPV	AC2 (95% CI)	F_1_-score
ChatGPT o3	0.65 (0.49–0.81)	0.82	0.98	0.62	0.93	0.97	0.53 (0.37–0.69)	0.87
Gemini Flash 2.0	0.47 (0.33–0.60)	0.75	0.93	0.69	0.94	0.95	0.52 (0.31–0.73)	0.83
DeepSeek v3	0.55 (0.34–0.75)	0.92	0.96	0.54	0.93	0.97	0.55 (0.35–0.74)	0.92
Grok 3	0.71 (0.60–0.83)	0.72	0.99	0.69	0.94	0.96	0.51 (0.32–0.69)	0.92

**Abbreviations:** AC2, Gwet second-order agreement coefficient; CI, confidence interval; Intra-κ, intraobserver Cohen kappa; κ, Cohen kappa; NPV, negative predictive value; PPV, positive predictive value.

**Table 4:** Performance of large language models on risk of bias 2 assessment metrics.

Model	Intra-κ (95% CI)	Sensitivity (High)	Sensitivity (High/some concerns)	Specificity	PPV	NPV	AC2 (95% CI)	F_1_-score
ChatGPT o3	0.48 (0.31–0.66)	0.55	0.96	0.78	0.38	0.48	0.17 (0.04–0.31)	0.45
Gemini Flash 2.0	0.52 (0.34–0.69)	0.05	0.47	0.99	0.5	0.61	0.45 (0.3–0.6)	0.09
DeepSeek v3	0.34 (0.14–0.54)	0.5	0.98	0.8	0.38	0.51	0.23 (0.09–0.37)	0.43
Grok 3	0.52 (0.35–0.69)	0.2	0.94	0.89	0.31	0.52	0.3 (0.17–0.43)	0.24

**Abbreviations:** AC2, Gwet second-order agreement coefficient; CI, confidence interval; Intra-κ, intraobserver Cohen kappa; κ, Cohen kappa; NPV, negative predictive value; PPV, positive predictive value.

For RoB 2, intraobserver reliability was lower than for RoB 1, with κ values ranging from 0.34 to 0.52. Sensitivity for identifying high-risk trials was modest (0.05–0.55), whereas specificity was higher (0.78–0.99). Positive predictive value (PPV) for high-risk judgements ranged from 0.31 to 0.50, while negative predictive value (NPV) ranged from 0.48 to 0.61. Thus, the models were limited in both ruling-in and ruling-out risk of bias. Sensitivity for identifying trials with any concern for bias (0.47–0.96) was higher, as most models tended to classify the majority of studies as having at least some concerns of bias. F_1_-scores (0.09–0.45) confirmed weak balance between precision and recall despite reasonable specificity. Stuart–Maxwell rejected equality of category proportions for all models (raw and BH-adjusted p < 0.001), indicating a systematic shift toward ‘Some concerns/High’ relative to the gold standard. Exact 95% confidence intervals for all RoB 2 diagnostic metrics are provided in eTable 5B, and domain-specific intra- and inter-observer κ values in eTable 7.

Visualisations of model performance, including a heatmap of agreement consistency for ChatGPT o3 and heatmaps of agreement with human ratings for all four models, are provided in eFig. 1 and eFig. 2, respectively.

### Sensitivity analysis

A sensitivity analysis addressed model limitations in extracting trial protocols by imputing gold-standard Domain 5 (Selective outcome reporting) values when protocols were unavailable, and recalculating Domain 6 (overall risk of bias). This did not improve overall RoB 2 performance metrics: Cohen κ remained steady at 0.05 (95% CI −0.07 to 0.18) for ChatGPT o3 and 0.19 (95% CI 0.02–0.36) for DeepSeek v3 (eTable 8). However, when LLMs rated “Some concerns” or “High bias” because trial protocol was not available, imputing gold standard value for Domain 5 improved interobserver reliability markedly. This indicates that low k for Domain 5 was largely dependent on failed protocol extraction. Highest k values were seen for DeepSeek v3 (0.96) and ChatGPT o3 (0.95). A heatmap of agreement with human ratings for all models after the sensitivity analysis is provided in eFig. 3 and domain-specific intra- and inter-observer κ values in eTable 9.

## Discussion

None of the evaluated LLM were reliable enough for independent machine-automated risk-of-bias judgement with either the RoB 1 or RoB 2 tools. For the Cochrane RoB 1 instrument, all models achieved fair agreement with human reviewers; DeepSeek v3 performed best but remained below the level typically reported for trained reviewers.^39^ For RoB2, interobserver reliability was substantially lower for RoB 2, with the highest κ being only 0.13 (Gemini Flash 2.0) but this is also typical to trained human reviewers who typically reach κ values of 0.15–0.20.^6^ Hence, the low reliability of models could in part be explained by the inherent variability among human consensus ratings in the gold standard. In addition to poor overall accuracy, models were limited in both ruling-in and ruling-out risk of bias, with PPV and NPV values indicating only modest discrimination.

For RoB 2, models rarely agreed with human assessors when the trial protocols were not available. All tested LLMs systemically failed to retrieve publicly available protocols even when prompted to retrieve them, and after correctly identifying registration numbers. When correct values for Domain 5 were imputed, interobserver reliability failed to improve, suggesting that discordance arose primarily from overly stringent classification rather than protocol retrieval failures. The performance of DeepSeek v3, a non-reasoning model, was unexpectedly positive, as it matched or exceeded reasoning-based models such as ChatGPT o3 and Grok 3. This result contrasts with recent AI benchmark tests, where reasoning-enabled (chain-of-thought) models generally demonstrate superior performance in complex reasoning tasks.^40^

Previous studies of automated RoB assessment have reported widely inconsistent findings. Best κ values were >0.8 when applied on a McMaster-modified Cochrane-based RoB-tool on an earlier version of ChatGPT. However, κ values as low as 0.2 have been published with a RoB 2 dataset on ChatGPT 4o (non-reasoning).^2^^,^^5^ A recent systematic review by Lieberum et al. highlighted the rapid growth of LLM-related publications in evidence synthesis, noting that many lacked methodological detail or used incorrect bias-assessment tools, with emphasis on the need for future high-quality validation studies conducted with strong methodological rigour.^41^ Our data extend these findings by demonstrating that modern LLMs, when prompted with full-text articles, attain fair reliability for RoB 1 and poor agreement for RoB 2. The diagnostic metrics further show that models distinguished levels of bias more effectively with RoB 1 than with RoB 2, suggesting that current LLMs manage simple categorical reasoning but struggle when contextual judgement is required for domain-based assessments.

Systematic reviewers increasingly screen thousands of studies. Given the models’ tendency to overclassify risk of bias and their limited ability to distinguish clearly high or low risk trials, their most suitable role is supervised triage, prioritising studies for human review or serving as a second assessor. In practice, an LLM could be used to screen all trials and flag those judged as high risk or with concerns of bias for full human assessment, while low-risk classifications are not accepted automatically but checked in a smaller random sample. Alternatively, the LLM could function as the second assessor in a dual-review workflow, with disagreements resolved by human consensus. Brief checking of excluded studies can reduce residual error, although the overall impact on workload will depend on how widely such models are adopted and integrated into review workflows.

Strengths of this study include (1) simultaneous testing of four modern LLMs, (2) full-text ingestion rather than abstract-only prompts, (3) preregistration, and (4) a sensitivity analysis that omits technical problems with the selective-reporting domain when protocols are not available. The main limitation is reliance on the gold standard reference, i.e., published RoB judgements that themselves may carry error. Trial selection was alphabetical and chronological rather than random, which may have introduced selection bias and may limit generalisability. For example, this approach may overrepresent certain investigators or trial reporting styles within each review or meta-analysis. Subgroup analyses by speciality or publication date were not performed, as the study was not powered for between-group comparisons and the number of trials per subgroup was limited. Trials were clustered within a small number of source reviews and meta-analyses, and human assessments within each cluster may be correlated. To address this, we performed a cluster bootstrap sensitivity analysis where cluster-adjusted confidence intervals were wider but it did not materially change results. Finally, κ down-weights agreement when one class dominates; thus, low κ for RoB 2 may overstate practical disagreement. The authors can also not exclude the possibility of data contamination, where the LLM might have been trained on the published meta-analyses used as gold reference. Assessment of LLM performance within specific specialities should be addressed in future studies.

Large-language models evolve on a rapid release cycle and continued progress is likely to improve their reliability in risk-of-bias assessment. Although current performance remains below the threshold required for autonomous use, advances in reasoning and contextual understanding may enhance their future accuracy. Three developments are likely to accelerate that progress: (1) fine-tuning models on clinical trial and medical literature databases to enhance domain-specific contextual reasoning; (2) automated retrieval of full trial protocols to reduce misclassification in selective-reporting domains, and (3) calibrated probability thresholds that balance sensitivity against reviewer workload. Prospective, in-process reviews are needed to confirm workload savings and to monitor model drift as these systems evolve.

In conclusion, among 100 RCTs across multiple clinical specialities, contemporary LLMs showed fair interobserver reliability for Cochrane RoB 1 screening and poor reliability for RoB 2. Although models performed better on simple categorical decisions, their limited ability to distinguish clearly high- or low-risk trials restricts autonomous use. At present, LLMs may assist reviewers through supervised triage or as secondary assessors to reduce reviewer workload. Domain-specific fine-tuning, automated protocol retrieval, and calibrated decision thresholds remain necessary before autonomous risk-of-bias assessment is feasible.

## Contributors

Lauri Nyrhi and Ilari Kuitunen had full access to all the data in the study and verified the accuracy of the data and the analyses. Lauri Nyrhi is the guarantor.

*Study concept and design:* Nyrhi, Kuitunen, Mattila, Ponkilainen, Karjalainen.

*Acquisition, analysis, or interpretation of data:* Nyrhi, Ponkilainen, Kuitunen, Laaksonen, Paljakka, Kuikka.

*Drafting of the manuscript:* Nyrhi, Ponkilainen, Kuitunen, Karjalainen, Mattila.

*Critical revision of the manuscript for important intellectual content:* Nyrhi, Ponkilainen, Kuitunen, Laaksonen, Paljakka, Kuikka, Mattila, Karjalainen.

*Statistical analysis:* Nyrhi, Kuitunen.

*Administrative, technical, or material support*: Nyrhi, Ponkilainen, Mattila, Karjalainen, Kuitunen.

*Study supervision:* Nyrhi, Ponkilainen, Mattila, Karjalainen, Kuitunen.

All authors read and approved the final version of the manuscript.

## Data sharing statement

All data and code used in this study are openly available in the OSF repository at https://doi.org/10.17605/OSF.IO/K45C7. These materials are released under a Creative Commons Attribution 4.0 International (CC BY 4.0) licence.

## Declaration of interests

No conflicts of interest exist.

## Appendix A Supplementary data

eFigures and eTables
