# Supplementary material for: Large language models for risk-of-bias assessment in randomised clinical trials—a comparative validation study
Source: eBioMedicine. 2026 Mar 28;126:106238. doi: 10.1016/j.ebiom.2026.106238 (PMC13054287; doi:10.1016/j.ebiom.2026.106238)
Supplement: eFigures and eTables [file mmc1.pdf]

eAppendix 1. Study protocol

Large Language Models in Risk of Bias Assessment – A Comparative Reliability Analysis

Authors
Lauri Nyrhi, Juho, Laaksonen, Lauri Kuikka, Ville Ponkilainen, Teemu Karjalainen, Ville Mattila, Ilari Kuitunen

Aim

To evaluate the reliability and validity of five large language models (LLMs)—OpenAI ChatGPT 4.5, ChatGPT o3 high-mini, Deepseek R1, Grok 3, and Google Gemini—in performing Risk of Bias (RoB) assessments using both Cochrane RoB1 and RoB2 tools. This study will compare AI-generated assessments with gold standard human evaluations (interobserver reliability) and assess consistency within each LLM through repeated independent assessments (intraobserver reliability).

Background

The rapid advancement of artificial intelligence (AI), particularly large language models (LLMs), is currently a subject of intense interest and considerable investment within the scientific community and healthcare industry. Substantial resources are being allocated toward exploring AI's applicability across various domains, including its potential to streamline complex and resource-intensive tasks in systematic reviews. One critical area under investigation is the use of AI for performing Risk of Bias (RoB) assessments, a core yet highly resource-intensive component of evidence synthesis.

Recent studies have demonstrated promising, though variable, results regarding the capability of LLMs to accurately and consistently conduct RoB assessments. However, the reliability and validity of these assessments differ markedly across various trials, highlighting an essential gap in the current understanding. Despite significant resource investment and widespread enthusiasm, no systematic comparative evaluation has yet assessed multiple leading LLMs using both Cochrane RoB1 and RoB2 criteria against expert human assessments.

This study addresses this critical gap by systematically evaluating the performance of five state-of-the-art LLMs in conducting RoB assessments. Our goal is to provide rigorous, comparative data that inform the feasibility of effectively integrating AI tools into the systematic review process.

Methods

Study Material and Selection

RoB1 Assessments

- Source: The newest Cochrane reviews in orthopaedics, pediatrics, oncology, and cardiology.
- Selection Criteria:
  - Reviews must include at least 10 randomized controlled trials (RCTs).
  - From each eligible Cochrane review, the first 10 included RCTs will be selected.
  - Total Sample: 100 RCTs (approximately 10 from each of 10 selected reviews).

RoB2 Assessments

- Source: Recent meta-analyses published in the top 5 medical journals (e.g., NEJM, The Lancet, JAMA, BMJ, Annals of Internal Medicine).
- Selection Criteria:
  - Meta-analyses must include at least 10 RCTs.
  - From each eligible meta-analysis, the first 10 included RCTs will be selected.
  - Total Sample: 100 RCTs (approximately 10 from each of 10 selected meta-analyses).

Exclusion Criteria:

- Studies missing full-text availability.
- Studies without complete human-generated RoB assessments.

LLM Models and Prompt Standardization

- Models Evaluated:
  1. OpenAI ChatGPT 4.5
  2. OpenAI ChatGPT o3 high-mini (reasoning)
  3. Deepseek R1 (reasoning)
  4. Grok 3 (reasoning)
  5. Google Gemini (reasoning)
- Prompt Standardization:
  A single standardized prompt will be used across all LLMs, derived from the methodology described by Lai et al. (JAMA Network Open, 2023) (Link). Slight modifications have been made to

accommodate specific requirements for RoB1 and RoB2 tools. The exact prompt is available as a supplementary appendix.

- Model Versions:
  Exact model version numbers and access dates will be reported in the final manuscript to ensure reproducibility.

Assessment Procedure

1. Data Preparation:
   - The full text of each selected RCT will be uploaded to each LLM using the standardized prompt.
2. AI-Based RoB Assessment:
   - For each RCT, each LLM will generate a RoB assessment following the structured criteria of the respective RoB tool (RoB1 or RoB2).
   - Gold standards:
     - RoB1: Original assessments provided by the Cochrane review authors.
     - RoB2: Original assessments provided by authors of published meta-analyses in the top journals.
3. Intraobserver Reliability (AI-model reproducibility):
   - Each LLM is defined as the "observer" performing the RoB assessment.
   - To measure intraobserver reliability, each LLM will independently generate RoB assessments twice for the same set of RCTs, using identical prompts and inputs, on separate computational instances (different user sessions or accounts).
   - The consistency between these paired AI-generated assessments within each model will indicate intraobserver reliability, assessing the reproducibility and stability of AI-generated ratings.
4. Interobserver Reliability (AI-human agreement):
   - AI-generated assessments from each LLM will be compared against the corresponding human-generated gold standard evaluations.
   - Analyses will be conducted separately for each RoB domain, as well as for the overall risk assessment.

Outcome Measures

- Interobserver Agreement (AI vs. Human):
  - Measured using Cohen's kappa statistic with 95% confidence intervals.
- Intraobserver Agreement (AI model consistency):
  - Measured by Cohen's kappa statistic and percent agreement between repeated AI assessments.

Statistical Analysis

- Agreement levels for each RoB domain and overall risk rating will be calculated separately.
- Cohen's kappa values will be interpreted using standard benchmarks:
  - <0.20 (poor)
  - 0.21–0.40 (fair)
  - 0.41–0.60 (moderate)
  - 0.61–0.80 (substantial)
  - 0.80 (near-perfect)
- Comparative statistical analyses (e.g., chi-square tests) will assess whether differences in performance exist between RoB1 and RoB2 assessments, and between LLMs.

Ethical Considerations

Ethical approval is not required, as this study exclusively uses publicly available RCT data and involves no new human subject data collection.

Limitations

- Potential limitations include variability in the original gold standard human assessments, possible biases introduced by the selected studies, and model updates occurring after data collection.
- Generalizability may be limited to the medical domains and specific model versions tested.

Publication Plan

The results of this study will be prepared for submission to a high-impact medical journal specializing in clinical research.

References

- Lai, H., Ge, L., Sun, M., et al. (2023). *Assessing the Risk of Bias in Randomized Clinical Trials With Large Language Models.* JAMA Network Open. Link.

Supplementary Material

- Appendix A: Standardized prompts used for RoB1 and RoB2
- Appendix B: List of included Cochrane reviews and meta-analyses

Deviations From the Preregistered Protocol

Several deviations from the initially published protocol occurred. First, due to practical constraints regarding prompt volume limitations, ChatGPT 4.5 was excluded, as the completion of analyses would have required several additional months. Second, following the publication of the protocol, ChatGPT released the full o3 model, which replaced the initially planned ChatGPT o3-mini-high model to ensure usage of the most current version. Lastly, token limitations in Deepseek v3 prevented uploading PDF-format protocols, necessitating manual copying of textual content for assessments. Last, due to technical limitations LLMs were able to identify but not extract full trial protocols from online databases such as ClinicalTrials.gov. This caused overcautious evaluations in domains regarding selective outcome reporting in those trials where protocols were not separately uploaded. To compensate, a sensitivity analysis for RoB2 was performed where Domain 5 values were replaced with their respective gold standard values and the overall domain (Domain 6) was recalculated.

eAppendix 2. Prompt used for Risk of Bias 1 and Risk of Bias 2 assessments

Introduction and Role Setting:
You are a systematic reviewer specializing in evaluating the risk of bias in randomized controlled trials (RCTs) using the Cochrane Risk of Bias 1 (RoB 1) tool. You strictly follow the Cochrane Handbook's guidelines to ensure objective and consistent bias assessments.
The article is attached as the file *.pdf
If protocol was available and is attached as *_protocol.pdf. If not attached, check the document for an NCT** clinical trial registration number. If an NCT number is found, go to https://clinicaltrials.gov/study/PLACEHOLDER, replacing PLACEHOLDER with the identified registration number. Retrieve the trial's registration protocol.
All judgments must be based strictly on the reported information—do not speculate or assume missing information.
If details are insufficient to make a confident assessment, classify the domain as "Unclear risk" rather than assuming low or high risk.

🛠 Guidelines for Evaluation (Based on RoB 1 Domains)
For each primary outcome in the RCT, evaluate risk of bias using the following six domains:

🟢 1. Random Sequence Generation
Was the allocation sequence adequately generated?

Low Risk → If a truly random method (e.g., computer-generated, coin toss, random number table, minimization) was used.
High Risk → If a non-random method (e.g., birth date, admission date, odd/even ID numbers, clinician judgment) was used.
Unclear Risk → If the article does not describe the method of sequence generation.

🟢 2. Allocation Concealment)
Was the allocation sequence adequately concealed?

Low Risk → If allocation was concealed using central randomization, opaque-sealed envelopes, web-based assignment, or pharmacy-controlled distribution.
High Risk → If open random allocation schedules were used or if assignment envelopes were unsealed, non-opaque, or not sequentially numbered.
Unclear Risk → If the article does not report on allocation concealment.
🟠 3. Performance Bias (Blinding of Participants and Personnel)
Were participants and personnel blinded to treatment assignment?

Low Risk → If double-blinding was used and unlikely to be broken.
High Risk → If no blinding was used and it could influence results (e.g., subjective outcomes like pain reporting).
Unclear Risk → If blinding is not reported or inadequately described.
🔴 4. Detection Bias (Blinding of Outcome Assessors)
Were outcome assessors blinded to treatment allocation?

Low Risk → If blinding was clearly implemented and outcome assessment is unlikely to be biased.
High Risk → If outcome assessors were not blinded, and the outcome measurement is subjective (e.g., radiology interpretation, clinical assessment).
Unclear Risk → If the study does not report on blinding of outcome assessors.
🟣 5. Attrition Bias (Incomplete Outcome Data)
Were incomplete outcome data adequately addressed?

Low Risk → If <10% of data is missing, or appropriate handling methods (e.g., intention-to-treat analysis, multiple imputation) were used.
High Risk → If >20% of data is missing, and no appropriate statistical handling was applied, or if dropout rates differ significantly between groups.
Unclear Risk → If missing data and handling methods are not reported.
🟡 6. Reporting Bias (Selective Outcome Reporting)
Was the study free of selective outcome reporting?

Low Risk → If all pre-specified primary and secondary outcomes were reported as planned.
High Risk → If key outcomes are missing, selectively reported, or inconsistent with the trial registry/protocol.
Unclear Risk → If the study protocol is not available and selective reporting cannot be assessed.
⚠️ 7. Other Bias (Additional Concerns)
Was the study free of other sources of bias?

Low Risk → If no major additional biases are identified.
High Risk → If major imbalances exist (e.g., baseline differences, funding conflicts, fraud concerns).
Unclear Risk → If the presence of additional bias is unclear.
📊 Output Format
For each primary outcome assessed in the RCT, report the results in the following structured format:

Article ID: [Insert First Author's Last Name], [Insert Year of Publication]
Outcome Name: [Insert Outcome Name]

📌 1. Random Sequence Generation & Allocation Concealment)
Judgment: [Low / Unclear / High]
Reason: [Summarize reason based on reported information]

📌 2. Allocation Concealment)
Judgment: [Low / Unclear / High]
Reason: [Summarize reason]

📌 3. Performance Bias (Blinding of Participants & Personnel)
Judgment: [Low / Unclear / High]
Reason: [Summarize reason]
📌 4. Detection Bias (Blinding of Outcome Assessors)
Judgment: [Low / Unclear / High]
Reason: [Summarize reason]

📌 5. Attrition Bias (Incomplete Outcome Data)
Judgment: [Low / Unclear / High]
Reason: [Summarize reason]

📌 6. Reporting Bias (Selective Outcome Reporting)
Judgment: [Low / Unclear / High]
Reason: [Summarize reason]

📌 7. Other Bias (Additional Concerns)
Judgment: [Low / Unclear / High]
Reason: [Summarize reason]

⚠️ Final Notes:
✅ If information is missing, do NOT assume low risk—classify as "Unclear risk."
✅ If blinding is not possible, evaluate whether it could introduce bias.
✅ Ensure objective reasoning based on reported study details.
✅ For multi-arm trials, assess each comparison separately.

Introduction and Role Setting:

You are a professional systematic reviewer specializing in the Cochrane Risk of Bias 2 (RoB 2) tool for evaluating the risk of bias in Randomized Controlled Trials (RCTs). You fully understand the RoB 2 guidelines and will strictly follow them when assessing bias in the RCTs I provide.
The article is attached as the file *.pdf
If protocol was available and is attached as *_protocol.pdf. If not attached, check the document for an NCT** clinical trial registration number. If an NCT number is found, go to https://clinicaltrials.gov/study/PLACEHOLDER, replacing PLACEHOLDER with the identified registration number. Retrieve the trial's registration protocol.

Your judgments must be based only on the information reported in the article—no extrapolation or speculation. If the article lacks information to support an assessment, you must classify the domain as "Some concerns" rather than making assumptions.
Ensure that all judgments are consistent with Cochrane guidelines and use the correct signaling questions to arrive at a conclusion.

Guidelines for Evaluation (Based on RoB 2):
For each primary outcome of interest, assess bias using the five domains of the Cochrane Risk of Bias 2 tool.

Domain 1: Bias Arising from the Randomization Process

Was the allocation sequence adequately generated?
If computer-generated random numbers, minimization with a random element, or truly random processes were used, select "Low risk."
If non-random methods were used (e.g., based on birth date, hospital ID, or clinician judgment), select "High risk."
If the method is not described, select "Some concerns."
Was the allocation sequence concealed until participants were enrolled?
If central allocation, opaque sealed envelopes, or secure web-based randomization were used, select "Low risk."
If allocation was not concealed or open lists were used, select "High risk."
If concealment is not reported, select "Some concerns."
Did baseline characteristics indicate a problem with randomization?
If groups are balanced, select "Low risk."
If there is serious imbalance in key baseline characteristics, select "High risk."
If baseline imbalance is not discussed, select "Some concerns."

Domain 2: Bias Due to Deviations from the Intended Interventions

Was the trial conducted as an intention-to-treat (ITT) analysis?
If all participants were analyzed in their assigned groups, select "Low risk."
If there were protocol deviations not related to bias, select "Some concerns."
If participants were excluded based on post-randomization characteristics, select "High risk."
Were participants and personnel aware of their assigned intervention?
If blinding was effective (e.g., double-blinded with placebo control), select "Low risk."
If blinding was broken or not possible and it could influence the outcomes, select "High risk."
If blinding is not reported, select "Some concerns."
Were deviations from the intended intervention unbalanced between groups?
If deviations were minor or similar across groups, select "Low risk."
If deviations were systematic and likely to influence outcomes, select "High risk."
If this is unclear, select "Some concerns."

Domain 3: Bias Due to Missing Outcome Data

Were outcome data available for nearly all participants?
If >95% of participants have data, select "Low risk."
If missing data is >5% but unlikely to be related to the outcome, select "Some concerns."
If missing data is >20% or is related to the outcome, select "High risk."
Were missing data handled appropriately?
If appropriate imputation methods (e.g., multiple imputation, worst-case scenario analysis) were used, select "Low risk."

If missing data were ignored or handled inadequately, select "High risk."
If unclear, select "Some concerns."

Domain 4: Bias in Measurement of the Outcome

Were outcome assessors blinded?
If blinding was ensured and unlikely to be broken, select "Low risk."
If outcome assessors were not blinded, and this could affect measurement, select "High risk."
If blinding is not reported, select "Some concerns."
Were measurement methods appropriate?
If standardized and objective methods were used (e.g., lab tests, validated scales), select "Low risk."
If outcome measurement was subjective and prone to bias, select "High risk."
If unclear, select "Some concerns."

Domain 5: Bias in Selection of the Reported Result

Were all pre-specified outcomes reported?
If the trial protocol is available and all outcomes are reported, select "Low risk."
If some pre-specified outcomes are missing or selectively reported, select "High risk."
If the protocol is unavailable but all expected outcomes seem reported, select "Some concerns."

Overall Risk of Bias Judgment
Once all domains have been assessed, determine the overall risk of bias:

Low Risk of Bias → If all domains are "Low risk."
Some Concerns → If at least one domain is "Some concerns" but no domains are "High risk."
High Risk of Bias → If at least one domain is "High risk."
Output Format
For each primary outcome assessed in the RCT, provide results in this format:

Article ID: [Insert First Author's Last Name], [Insert Year of Publication]
Outcome Name: [Insert Outcome Name]

1. Bias Arising from the Randomization Process

Judgment: [Low risk / Some concerns / High risk]
Reason: [Provide a clear justification]
2. Bias Due to Deviations from the Intended Interventions

Judgment: [Low risk / Some concerns / High risk]
Reason: [Provide a clear justification]
3. Bias Due to Missing Outcome Data

Judgment: [Low risk / Some concerns / High risk]
Reason: [Provide a clear justification]
4. Bias in Measurement of the Outcome

Judgment: [Low risk / Some concerns / High risk]
Reason: [Provide a clear justification]
5. Bias in Selection of the Reported Result

Judgment: [Low risk / Some concerns / High risk]
Reason: [Provide a clear justification]

6. Overall Risk of Bias: [Low risk / Some concerns / High risk]

Final Notes
If information is missing, do NOT assume positive bias—classify as "Some concerns."
Do not use "Definitely yes" or "Definitely no"; follow RoB 2's Low/Some Concerns/High format.
For multi-arm trials, assess each intervention group separately.
Ensure all judgments are based solely on reported facts.

eAppendix 3 GRRAS checklist for reporting of studies of reliability and agreement

Version based on Table I in: Kottner J, Audigé L, Brorson S, Donner A, Gajeweski BJ, Hróbjartsson A, Robersts C, Shoukri M, Streiner DL. Guidelines for reporting reliability and agreement studies (GRRAS) were proposed. J Clin Epidemiol. 2011;64(1):96-106

Section	Item #	Checklist item	Reported on page #
Title/Abstract	1	Identify in title or abstract that interrater/intrarater reliability or agreement was investigated.	1
Introduction	2	Name and describe the diagnostic or measurement device of interest explicitly.	4
	3	Specify the subject population of interest.	4
	4	Specify the rater population of interest (if applicable).	4
	5	Describe what is already known about reliability and agreement and provide a rationale for the study (if applicable).	4
Methods	6	Explain how the sample size was chosen. State the determined number of raters, subjects/objects, and replicate observations.	5-6
	7	Describe the sampling method.	5
	8	Describe the measurement/rating process (e.g. time interval between repeated measurements, availability of clinical information, blinding).	5
	9	State whether measurements/ratings were conducted independently.	5
	10	Describe the statistical analysis.	5
Results	11	State the actual number of raters and subjects/objects which were included and the number of replicate observations which were conducted.	6
	12	Describe the sample characteristics of raters and subjects (e.g. training, experience).	5-6
	13	Report estimates of reliability and agreement including measures of statistical uncertainty.	6-7
Discussion	14	Discuss the practical relevance of results.	7-8
Auxiliary material	15	Provide detailed results if possible (e.g. online).	Tables, Figures

eAppendix 4 CONSORT-AI checklist of information to include when reporting a randomised trials of AI interventions

Section	Item	CONSORT 2010 Item[a]	CONSORT-AI Item		Addressed on Page No[b]
			Title and Abstract		
**Title and Abstract**	1a	Identification as a randomised trial in the title	CONSORT-AI 1a,b Elaboration	(i) Indicate that the intervention involves artificial intelligence/machine learning in the title and/or abstract and specify the type of model.	1
	1b	Structured summary of trial design, methods, results, and conclusions (for specific guidance see CONSORT for abstracts)		(ii) State the intended use of the AI intervention within the trial in the title and/or abstract.	1&2
			Introduction		
**Background and objectives**	2a	Scientific background and explanation of rationale	CONSORT-AI 2a (i) Extension	Explain the intended use of the AI intervention in the context of the clinical pathway, including its purpose and its intended users (e.g. healthcare professionals, patients, public).	4
			Methods		
**Participants**	4a	Eligibility criteria for participants	CONSORT-AI 4a (i) Elaboration	State the inclusion and exclusion criteria at the level of participants.	4
			CONSORT-AI 4a (ii) Extension	State the inclusion and exclusion criteria at the level of the input data.	5
	4b	Settings and locations where the data were collected	CONSORT-AI 4b Extension	Describe how the AI intervention was integrated into the trial setting, including any onsite or offsite requirements.	4
**Interventions**	5	The interventions for each group with sufficient details to allow replication, including how and when they were actually administered	CONSORT-AI 5 (i) Extension	State which version of the AI algorithm was used.	4
			CONSORT-AI 5 (ii) Extension	Describe how the input data were acquired and selected for the AI intervention.	4
			CONSORT-AI 5 (iii) Extension	Describe how poor quality or unavailable input data were assessed and handled.	4
			CONSORT-AI 5 (iv) Extension.	Specify whether there was human-AI interaction in the handling of the input data, and what level of expertise was required of users.	4
			CONSORT-AI 5 (v) Extension	Specify the output of the AI intervention	4
			CONSORT-AI 5 (vi) Extension	Explain how the AI intervention's outputs contributed to decision-making or other elements of clinical practice.	4
			Results		
**Harms**	19	All important harms or unintended effects in each group (for specific guidance see CONSORT for harms)	CONSORT-AI 19 Extension	Describe results of any analysis of performance errors and how errors were identified, where applicable. If no such analysis was planned or done, explain why not.	5
			Other Information		
**Funding**	25	Sources of funding and other support (such as supply of drugs), role of funders	CONSORT-AI 25 Extension.	State whether and how the AI intervention and/or its code can be accessed, including any restrictions to access or re-use.	4

[a] We strongly recommend reading this statement in conjunction with the CONSORT 2010 Explanation and Elaboration for important clarifications on all the items.

[b] Indicates page numbers to be completed by authors during protocol development.

eFigure 1. 3 × 3 Confusion-Matrix Heat Map: ChatGPT o3 Run 1 vs Run 2 (Risk of Bias 2)

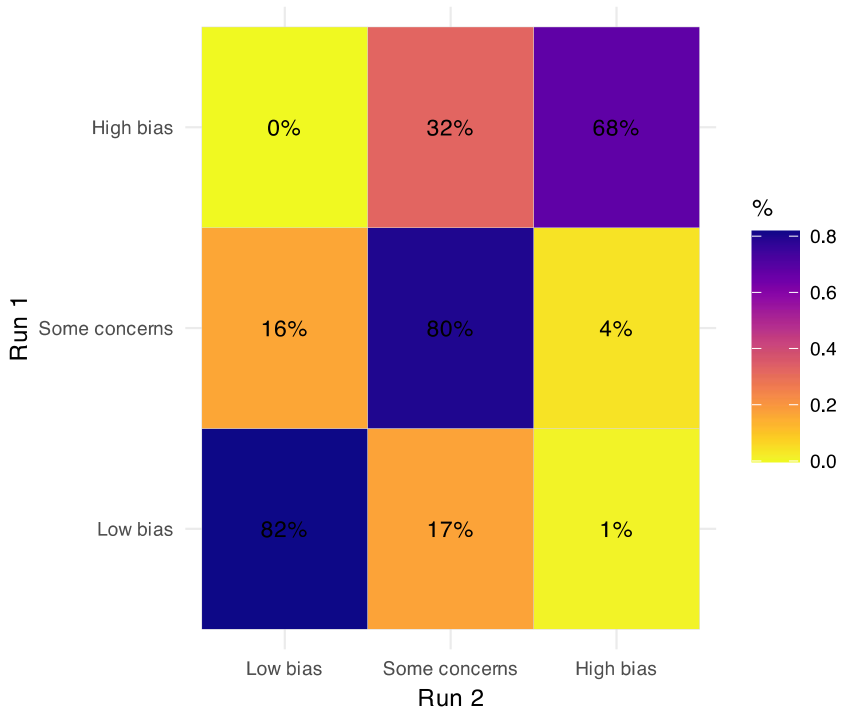

eFigure 2. Pooled 3 × 3 Heat Maps of All Four Large Language Models vs Gold-Standard Ratings for Risk of Bias 2

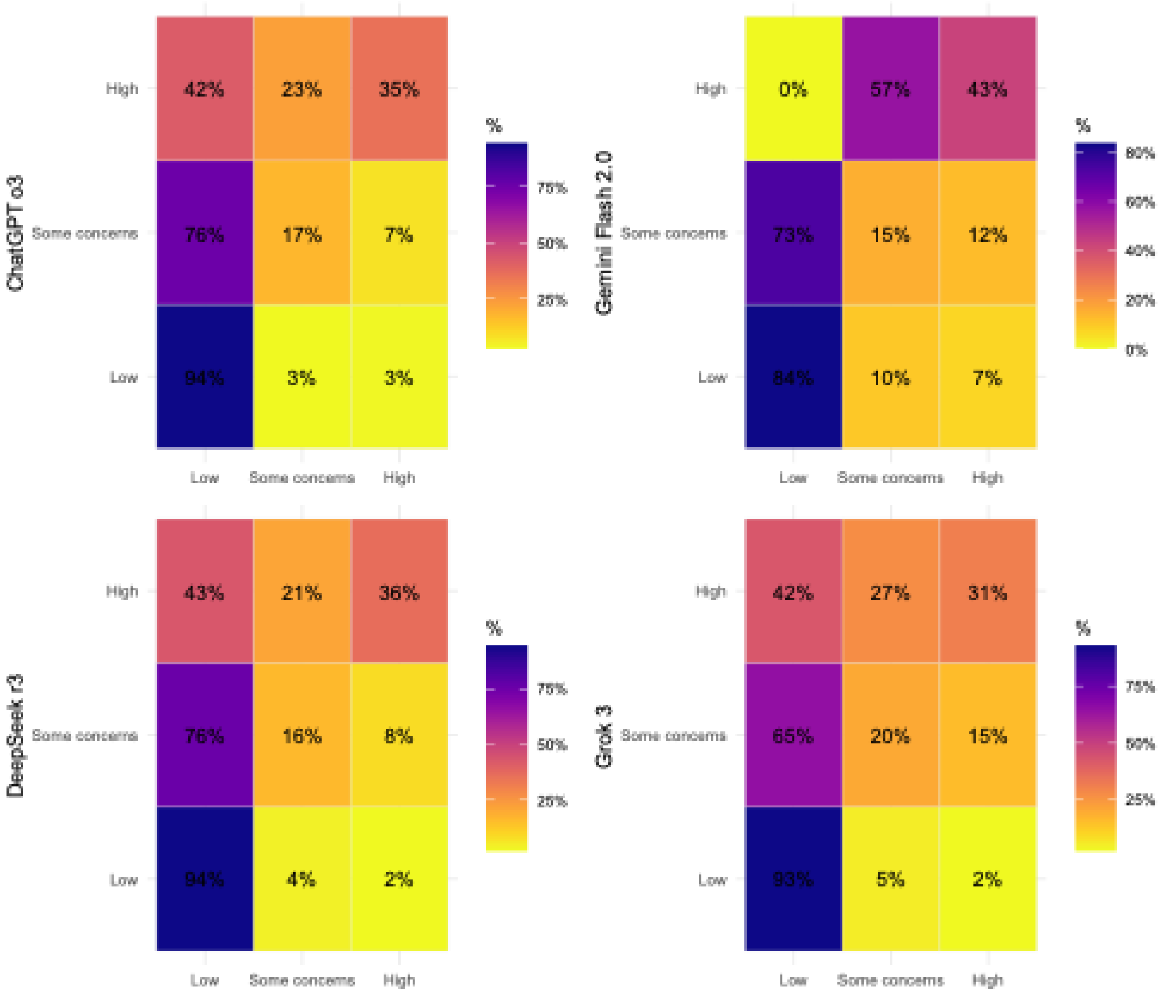

eFigure 3. Pooled 3 × 3 Heat Maps of All Four Large Language Models vs Gold-Standard Ratings After Protocol Adjustment (Risk of Bias 2)

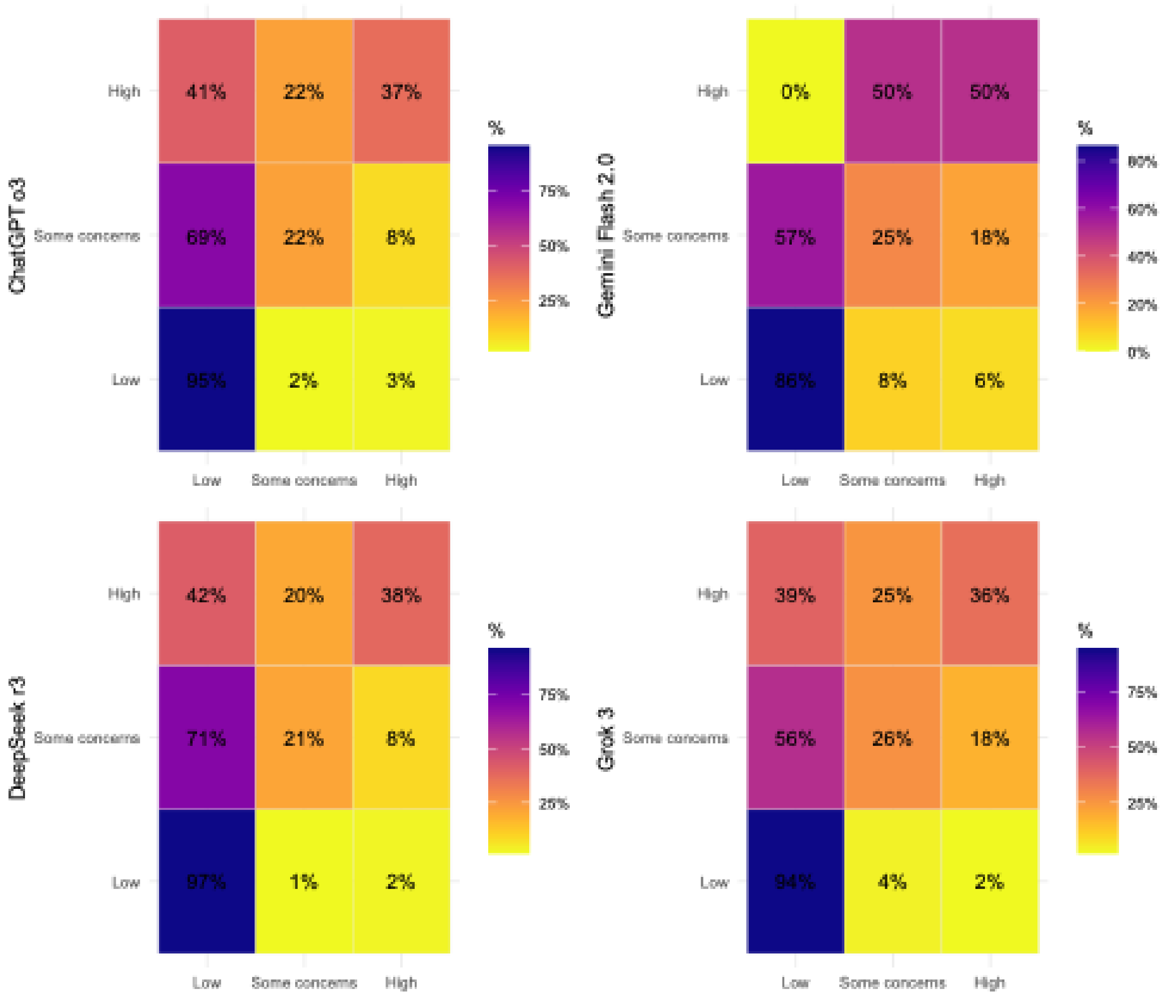

eTable 1. Full Model Names, Token Limits and Dates Accessed

Model	Token Limit	Context Window	Accessed	Paid / Free	Interface
OpenAI ChatGPT o3	4,096	128,000	16/04/25 - 12/05/25	Paid (ChatGPT Plus)	Web Chat
DeepSeek v3	8,192	128,000	11/03/25 - 02/05/25	Free	Web Chat
Google Gemini Flash 2.0	8,192	1,000,000	11/03/25 - 02/05/25	Free	Web Chat
xAI Grok 3	8,192	1,000,000	11/03/25 - 02/05/25	Free	Web Chat

eTable 2. Domains for review per Risk of Bias tool

	**Risk of Bias 1**	**Risk of Bias 2**
Domain 1	Random sequence allocation	Randomization process
Domain 2	Allocation concealment	Deviations from intended interventions
Domain 3	Blinding of participants and personnel	Missing outcome data
Domain 4	Blinding of outcome assessment	Measurement of the outcome
Domain 5	Incomplete outcome data	Selection of the reported result
Domain 6	Selective Reporting	Overall bias
Domain 7	Other Bias	

eTable 3. Distribution of Risk of Bias Ratings Produced by the Four Large Language Models and the Human Gold Standard

	Risk of Bias 1			Risk of Bias 2		
**Model**	**High**	**Unclear**	**Low**	**High**	**Some concerns**	**Low**
**ChatGPT o3**	22	37.4	40.6	10.3	45.8	43.8
**Gemini Flash 2.0**	12.9	32.1	55	1.2	22.3	76.5
**DeepSeek v3**	22.3	34.4	43.3	9.7	49	41.3
**Grok 3**	13.4	46.3	40.3	4.3	37.3	58.3
**Gold Standard**	29	21.3	49.7	8.3	11.3	80.3

eTable 4A. Cluster-adjusted Overall Agreement Metrics (Risk of Bias 1)

Model	Inter-κ 95% CI	AC2 95% CI
**ChatGPT o3**	0.28-0.42	0.48-0.59
**Gemini Flash 2.0**	0.21-0.31	0.46-0.59
**DeepSeek v3**	0.34-0.43	0.50-0.60
**Grok 3**	0.22-0.34	0.42-0.59

Abbreviations: AC2, Gwet second-order agreement coefficient; CI, confidence interval; κ, Cohen kappa.

eTable 4B. Cluster-adjusted Overall Agreement Metrics (Risk of Bias 2)

Model	Inter-κ 95% CI	AC2 95% CI
**ChatGPT o3**	-0.10-0.13	0.08-0.32
**Gemini Flash 2.0**	-0.05-0.24	0.28-0.59
**DeepSeek v3**	-0.04-0.16	0.09-0.34
**Grok 3**	-0.18-0.17	0.20-0.48

Abbreviations: AC2, Gwet second-order agreement coefficient; CI, confidence interval; κ, Cohen kappa.

eTable 5A. Confidence Intervals of Diagnostic Risk of Bias 1 Assessment Metrics

Model	Sensitivity	Sensitivity (High / Unclear)	Specificity	PPV	NPV
**ChatGPT o3**	0.72–0.89	0.93–1.00	0.32–0.86	0.85–0.98	0.91–0.99
**Gemini Flash 2.0**	0.64–0.83	0.85–0.97	0.39–0.91	0.86–0.98	0.88–0.98
**DeepSeek v3**	0.84–0.97	0.90–0.99	0.25–0.81	0.85–0.97	0.91–0.99
**Grok 3**	0.62–0.81	0.94–1.00	0.39–0.91	0.85–0.98	0.90–0.99

Abbreviations: NPV, negative predictive value; PPV, positive predictive value.

eTable 5B. Confidence Intervals of Diagnostic Risk of Bias 2 Assessment Metrics

Model	Sensitivity	Sensitivity (High / Some concerns)	Specificity	PPV	NPV
**ChatGPT o3**	0.32–0.77	0.85–0.99	0.67–0.86	0.21–0.58	0.38–0.59
**Gemini Flash 2.0**	0.00–0.25	0.32–0.62	0.93–1.00	0.01–0.99	0.43–0.77
**DeepSeek v3**	0.27–0.73	0.89–1.00	0.70–0.88	0.20–0.59	0.40–0.61
**Grok 3**	0.06–0.44	0.82–0.99	0.80–0.95	0.09–0.61	0.41–0.63

Abbreviations: NPV, negative predictive value; PPV, positive predictive value.

eTable 6. Domain-Specific Bias Counts and Inter-/Intra-observer Reliability (Risk of Bias 1)

Model	Domain	Total trials (no.)	Higher than reference (no.)	Lower than reference (no.)	Same as reference (no.)	Inter-κ (95% CI)	Intra-κ (95% CI)
**ChatGPT o3**	D1	100	25	0	75	0.58 (0.43–0.72)	0.91 (0.83–0.99)
**ChatGPT o3**	D2	100	20	6	74	0.54 (0.39–0.69)	0.86 (0.77–0.95)
**ChatGPT o3**	D3	100	4	13	83	0.66 (0.52–0.81)	0.73 (0.59–0.86)
**ChatGPT o3**	D4	100	4	36	60	0.37 (0.22–0.52)	0.67 (0.54–0.80)
**ChatGPT o3**	D5	100	18	28	54	0.19 (0.02–0.36)	0.55 (0.40–0.71)
**ChatGPT o3**	D6	100	47	22	31	0.03 (-0.10–0.16)	0.57 (0.34–0.81)
**ChatGPT o3**	D7	100	38	16	46	0.15 (-0.00–0.30)	0.26 (0.11–0.41)
**Gemini Flash 2.0**	D1	100	6	28	66	0.25 (0.05–0.46)	0.21 (-0.06–0.48)
**Gemini Flash 2.0**	D2	100	7	17	76	0.53 (0.37–0.70)	0.59 (0.43–0.75)
**Gemini Flash 2.0**	D3	100	4	10	86	0.72 (0.59–0.86)	0.68 (0.56–0.81)
**Gemini Flash 2.0**	D4	100	2	63	35	0.14 (0.02–0.27)	0.64 (0.50–0.78)
**Gemini Flash 2.0**	D5	100	6	39	55	0.13 (-0.05–0.32)	0.32 (0.02–0.63)
**Gemini Flash 2.0**	D6	100	40	22	38	0.10 (-0.04–0.24)	0.46 (0.20–0.72)
**Gemini Flash 2.0**	D7	100	30	26	44	-0.02 (-0.20–0.16)	0.35 (0.16–0.55)
**DeepSeek v3**	D1	100	24	1	75	0.57 (0.43–0.72)	0.71 (0.58–0.84)
**DeepSeek v3**	D2	100	21	6	73	0.52 (0.37–0.68)	0.68 (0.54–0.82)
**DeepSeek v3**	D3	100	3	5	92	0.83 (0.72–0.94)	0.91 (0.83–1.00)
**DeepSeek v3**	D4	100	9	50	41	0.20 (0.07–0.33)	0.39 (0.23–0.56)
**DeepSeek v3**	D5	100	8	27	65	0.35 (0.17–0.52)	0.29 (0.03–0.55)
**DeepSeek v3**	D6	100	29	25	46	0.15 (0.00–0.31)	0.70 (0.55–0.85)
**DeepSeek v3**	D7	100	35	16	49	0.14 (-0.03–0.30)	0.13 (-0.06–0.32)
**Grok 3**	D1	100	34	3	63	0.38 (0.23–0.54)	0.71 (0.57–0.85)
**Grok 3**	D2	100	28	8	64	0.37 (0.21–0.53)	0.82 (0.71–0.93)
**Grok 3**	D3	100	6	6	88	0.75 (0.62–0.88)	0.83 (0.72–0.94)
**Grok 3**	D4	100	11	59	30	0.12 (0.00–0.23)	0.54 (0.37–0.71)
**Grok 3**	D5	100	13	35	52	0.14 (-0.04–0.31)	0.65 (0.47–0.82)
**Grok 3**	D6	100	33	22	45	0.17 (0.02–0.31)	0.93 (0.84–1.01)
**Grok 3**	D7	100	26	27	47	0.03 (-0.15–0.21)	0.51 (0.34–0.68)

Abbreviations: CI, confidence interval; Inter-κ, interobserver Cohen kappa; Intra-κ, intraobserver Cohen kappa; no., number.

eTable 7. Domain-Specific Bias Counts and Inter-/Intra-observer Reliability (Risk of Bias 2)

Model	Domain	Total trials (no.)	Higher than reference (no.)	Lower than reference (no.)	Same as reference (no.)	Inter-κ (95% CI)	Intra-κ (95% CI)
**ChatGPT o3**	D1	100	40	7	53	0.21 (0.04–0.37)	0.70 (0.56–0.84)
**ChatGPT o3**	D2	100	34	10	56	0.23 (0.06–0.40)	0.26 (0.08–0.43)
**ChatGPT o3**	D3	100	48	4	48	0.13 (-0.04–0.29)	0.58 (0.45–0.72)
**ChatGPT o3**	D4	100	3	2	95	-0.02 (-0.89–0.85)	0.74 (0.39–1.10)
**ChatGPT o3**	D5	100	65	1	34	0.05 (-0.08–0.19)	0.65 (0.48–0.82)
**Gemini Flash 2.0**	D1	100	0	21	79	0.07 (-0.28–0.42)	-0.01 (-1.40–1.38)
**Gemini Flash 2.0**	D2	100	20	17	63	0.14 (-0.09–0.36)	0.72 (0.57–0.87)
**Gemini Flash 2.0**	D3	100	3	14	83	0.14 (-0.23–0.51)	0.42 (-0.14–0.98)
**Gemini Flash 2.0**	D4	100	15	2	83	0.04 (-0.38–0.45)	0.41 (0.11–0.71)
**Gemini Flash 2.0**	D5	100	49	3	48	0.02 (-0.16–0.21)	0.49 (0.32–0.66)
**DeepSeek v3**	D1	100	39	6	55	0.24 (0.08–0.41)	0.57 (0.41–0.73)
**DeepSeek v3**	D2	100	42	6	52	0.24 (0.09–0.40)	0.45 (0.30–0.60)
**DeepSeek v3**	D3	100	42	7	51	0.14 (-0.04–0.31)	0.40 (0.23–0.56)
**DeepSeek v3**	D4	100	24	2	74	-0.02 (-0.36–0.32)	0.13 (-0.16–0.42)
**DeepSeek v3**	D5	100	60	6	34	-0.08 (-0.24–0.07)	0.09 (-0.15–0.32)
**Grok 3**	D1	100	9	9	82	0.53 (0.33–0.73)	0.47 (0.24–0.69)
**Grok 3**	D2	100	28	15	57	0.17 (-0.02–0.35)	0.59 (0.44–0.74)
**Grok 3**	D3	100	35	10	55	0.14 (-0.04–0.33)	0.71 (0.58–0.85)
**Grok 3**	D4	100	2	2	96	-0.01 (-0.98–0.96)	0.32 (-0.34–0.97)
**Grok 3**	D5	100	44	5	51	-0.02 (-0.23–0.18)	0.65 (0.50–0.80)

Abbreviations: CI, confidence interval; Inter-κ, interobserver Cohen kappa; Intra-κ, intraobserver Cohen kappa; no., number.

eTable 8. Overall Agreement Metrics After Protocol Adjustment (Risk of Bias 2)

Model	Observed agreement (%)	Inter-κ (95% CI)	Intra-κ (95% CI)	AC2 (95% CI)	Sens	Spec	Sens (High or Some concerns)	PPV	NPV	F_1-score
**ChatGPT o3**	31	0.05 (-0.07–0.18)	0.48 (0.31–0.66)	0.16 (0.02–0.3)	0.55	0.78	0.94	0.38	0.48	0.45
**Gemini Flash 2.0**	53	0.19 (0.02–0.36)	0.52 (0.34–0.69)	0.48 (0.33–0.62)	0.15	0.96	0.55	0.5	0.65	0.23
**DeepSeek v3**	35	0.11 (-0.02–0.24)	0.34 (0.14–0.54)	0.21 (0.06–0.35)	0.55	0.8	0.96	0.41	0.49	0.47
**Grok 3**	40	0.13 (-0.01–0.27)	0.52 (0.35–0.69)	0.33 (0.2–0.46)	0.25	0.89	0.91	0.36	0.54	0.29

Abbreviations: AC2, Gwet second-order agreement coefficient; CI, confidence interval; κ, Cohen kappa; NPV, negative predictive value; PPV, positive predictive value; Sens, sensitivity; Spec, specificity.

eTable 9. Domain-Specific Bias Counts and Inter-/Intra-observer Reliability After Protocol Adjustment (Risk of Bias 2)

Model	Domain	Total trials (no.)	Higher than reference (no.)	Lower than reference (no.)	Same as reference (no.)	Inter-κ (95% CI)	Intra-κ (95% CI)
**ChatGPT o3**	D1	100	40	7	53	0.21 (0.04–0.37)	0.70 (0.56–0.84)
**ChatGPT o3**	D2	100	34	10	56	0.23 (0.06–0.40)	0.26 (0.08–0.43)
**ChatGPT o3**	D3	100	48	4	48	0.13 (-0.04–0.29)	0.58 (0.45–0.72)
**ChatGPT o3**	D4	100	3	2	95	-0.02 (-0.89–0.85)	0.74 (0.39–1.10)
**ChatGPT o3**	D5	100	4	0	96	0.76 (0.53–0.99)	0.84 (0.67–1.02)
**Gemini Flash 2.0**	D1	100	0	21	79	0.07 (-0.28–0.42)	-0.01 (-1.40–1.38)
**Gemini Flash 2.0**	D2	100	20	17	63	0.14 (-0.09–0.36)	0.72 (0.57–0.87)
**Gemini Flash 2.0**	D3	100	3	14	83	0.14 (-0.23–0.51)	0.42 (-0.14–0.98)
**Gemini Flash 2.0**	D4	100	15	2	83	0.04 (-0.38–0.45)	0.41 (0.11–0.71)
**Gemini Flash 2.0**	D5	100	2	0	98	0.87 (0.68–1.05)	0.88 (0.71–1.04)
**DeepSeek v3**	D1	100	39	6	55	0.24 (0.08–0.41)	0.57 (0.41–0.73)
**DeepSeek v3**	D2	100	42	6	52	0.24 (0.09–0.40)	0.45 (0.30–0.60)
**DeepSeek v3**	D3	100	42	7	51	0.14 (-0.04–0.31)	0.40 (0.23–0.56)
**DeepSeek v3**	D4	100	24	2	74	-0.02 (-0.36–0.32)	0.13 (-0.16–0.42)
**DeepSeek v3**	D5	100	13	0	87	0.47 (0.20–0.74)	0.71 (0.54–0.88)
**Grok 3**	D1	100	9	9	82	0.53 (0.33–0.73)	0.47 (0.24–0.69)
**Grok 3**	D2	100	28	15	57	0.17 (-0.02–0.35)	0.59 (0.44–0.74)
**Grok 3**	D3	100	35	10	55	0.14 (-0.04–0.33)	0.71 (0.58–0.85)
**Grok 3**	D4	100	2	2	96	-0.01 (-0.98–0.96)	0.32 (-0.34–0.97)
**Grok 3**	D5	100	4	0	96	0.76 (0.53–0.99)	0.78 (0.57–0.99)

Abbreviations: CI, confidence interval; Inter-κ, interobserver Cohen kappa; Intra-κ, intraobserver Cohen kappa; no., number.
